# Is a Good Sleep on Mosquito-Free Nights Worth the Risk of Lymphoma Associated with the Use of Household Insecticides? A Case-Control Study of Lymphoma Subtypes in Adults

**DOI:** 10.3390/toxics11090752

**Published:** 2023-09-05

**Authors:** Pierluigi Cocco, Giannina Satta, Valerio Cancedda, Federico Meloni, Simone Milia, Ilaria Pilia, Mariagrazia Zucca, Sara De Matteis

**Affiliations:** 1 Centre for Occupational and Environmental Health, Division of Population Studies, Healthcare Research & Primary Care, Faculty of Health Sciences, University of Manchester, Manchester M13 9PL, UK; 2 Department of Medical Sciences and Public Health, University of Cagliari, 09131 Cagliari, Italy; gianninasatta@tiscali.it (G.S.); valposta@gmail.com (V.C.); federicomeloni@hotmail.it (F.M.); simonemilia.dr@gmail.com (S.M.); drssa.pilia@gmail.com (I.P.); sara.dem@unica.it (S.D.M.); 3 Unit of Laboratory Medicine, Sulcis Local Health Unit, 09013 Carbonia, Italy; mariagrazia.zucca@aslsulcis.it

**Keywords:** case-control study, multiple myeloma, DDT, propoxur, pyretroids

## Abstract

Background. The evidence linking the use of household pesticides and the risk of lymphoma is scanty. Methods. We explored the hypothesis in a population-based case-control study on lymphoma conducted in Sardinia, Italy, in 1998–2004, including 325 cases and 465 population controls and data on lifetime frequency, seasonality, and years of use of household insecticides and potential confounders. We calculated the risk of lymphoma (all subtypes) and its major subtypes associated with using household insecticides in three time windows (up to 1978, from 1979–2001, and 2002 onwards) with unconditional logistic regression adjusting by age, sex, education, and occupational exposure to pesticides. Results. Household insecticides did not increase risk of lymphoma (all subtypes), Hodgkin’s lymphoma, B-cell lymphoma, and the major B-cell lymphoma subtypes. The risk of multiple myeloma (MM) but not the other subtypes showed a non-significant upward trend (*p* = 0.203) with increasing quartiles of days of use in the time window when propoxur was the most popular household insecticide. Conclusions. Our results suggest no association between the household use of insecticides and the risk of lymphoma. Further studies are warranted to confirm or discard an association between MM risk and the use of propoxur.

## 1. Introduction

Although exposure to chemicals and, particularly, pesticides and solvents has emerged in the last decades as a contributing factor, along with viral, lifestyle and genetic polymorphisms, the aetiology of lymphomas remains unclear [1]. In 1991, the International Agency for Research on Cancer (IARC) classified occupational spraying and applying of insecticides as probably carcinogenic to humans (group 2A) because of limited evidence from epidemiological studies; adult lymphoma was one of the cancer sites suspected of an association [2]. The IARC classification of human carcinogens also includes several insecticides; however, only lindane, an obsolete organochlorine, was classified in Group 1 (carcinogenic to humans) and dichlorodiphenyltrichloroethane (DDT), diazinon, and malathion were listed as probable human carcinogens (Group 2A) [3]. In all these instances, the final evaluation indicated non-Hodgkin’s lymphoma (NHL) as the associated neoplastic outcome, alone or together with a few others [4,5]. NHL includes a group of neoplastic diseases arising from lymphocytes at different stages of maturation. Each subtype shows a peculiar immunophenotypic profile and its own range of epidemiological associations [1]. Disentangling the relevant specific agent from among a myriad of different molecules used for preventive and curative treatments of different crops in association with specific disease entities with enough statistical power is one of the most challenging tasks epidemiology faces nowadays [6]. The relative paucity of agrochemicals in the IARC groups 1 or 2A reflects such difficulties.

Nonetheless, a few studies explored in more detail the complex association between lymphoma and exposure to insecticides. In a pooled analysis of a large European population- based case-control study, occupational exposure to organophosphates showed an association with the risk of chronic lymphocytic leukaemia (CLL) [7]. On the other hand, Zheng et al. observed an excess NHL risk among farmers exposed to carbamate insecticides and, particularly, Sevin [8]. A meta-analysis showed that NHL as a whole and specific B-cell lymphoma subtypes were associated with occupational exposure to insecticides, such as organochlorines, organophosphates, and carbamates [9]; in particular, diffuse large B cell lymphoma (DLBCL), CLL, follicular lymphoma (FL), and T cell lymphoma were associated with use of carbamates, organophosphates, and organochlorines in one or more studies. The largest study on the agricultural use of insecticides and risk of NHL subtypes was the pooled analysis of the work history of 7909 cases and 8644 controls from nine international case-control studies contributing to the InterLymph Consortium [10]. Ever exposure to diazinon, a widely used organophosphate insecticide, and carbaryl, the best-known carbamate insecticide, was associated with a 30% increase in the risk of NHL (all subtypes combined), further raising to 2-fold for exposures lasting 8 years or more. Among the NHL subtypes, FL risk was elevated for ever use of organochlorine and organophosphate insecticides, with the strongest associations observed with diazinon (OR = 2.05, 95% CI 1.24–3.37) and carbaryl (OR = 1.89, 95% CI 1.23–2.90). Risk of the T-cell lymphoma subgroup was also linked to carbamate insecticides and, specifically, carbaryl [10]. However, in this large study, authors could not explore trends by exposure metrics other than years of use.

While the overall epidemiological evidence of the association between occupational exposure to specific insecticides and the risk of a few lymphoma subtypes seems consistent, only a few studies explored the association with residential exposure resulting from proximity to the crop fields and household use of insecticides. A U.S. study showed that environmental exposure to agrochemicals decreased with distance from crop fields among rural residents [11]. Some case-control studies addressed the risk of malignancies of the haemolymphatic system among children associated with exposure to household insecticides; both extermination of insects by professional pest controllers (OR = 2.6, 95% CI: 1.2–5.7) and frequency of parental use of household insecticides (*p* = 0.02) were associated with an increase in risk of childhood NHL [12]. Also, the household use of the carbamate insecticide propoxur was linked with risk of childhood leukemia [13]. Among adults, the risk of NHL increased with level of chlordane, used in the household against termites, measured in the carpet dust [14]. Cumulative exposure to household pesticides was also associated with NHL risk in another U.S. study [15]. Risk was more accentuated among women who applied themselves the product, for use of mothballs, and for exposures starting in 1950–1969 [15]. A large prospective cohort of U.S. women found a moderate association between insecticide use and the risk of NHL and, especially, its subtypes DLBCL and the combination of CLL and small lymphocytic lymphoma (CLL/SLL).

A generic exposure definition, the reliance on years of use to characterize exposure, lack of information to reconstruct exposure levels and dose-response trends, the frequent use of the generic diagnosis NHL, and the small number of cases of specific NHL subtypes make the results difficult to interpret. Besides, before drawing any conclusion, confounders, such as socioeconomic factors, dust, sunlight, or infectious agents, need to be ruled out [16].

The global market of household insecticides has been increasing by around 7% over the years and, expectedly, will keep growing in the foreseeable future. The increasing temperatures and the expansion northward of the areas covered by vector-borne insects due to climate change are major drivers of such upward trends [17]. On the other hand, indoor use of insecticides contributes to poor air quality and can be detrimental to human health. Over the years, the increasing pressure by regulatory agencies has led to shifting towards less toxic, less persistent products. However, in some parts of the world, use of persistent organochlorine insecticides continues for antimalarial purposes. For instance, DDT concentrations of 3.9 μg/m^3^ in the air and 1.2 mg/m^2^ in the floor dust were detected in specific areas of South Africa where indoor residual spray (IRS) was in use to control malaria vectors [18]. It is plausible that the past use of DDT in developed countries might have resulted in similar concentrations. Indoor spraying of propoxur resulted in air levels above 5 mg/m^3^, followed by constant decay and a 95% removal within 40 min [19]. Ventilation reduced the concentrations and accelerated the removal of the insecticide. In comparison, spraying pyrethrins resulted in lower air concentrations and almost complete removal after 5 min [19]. The allethrin concentration in indoor air samples generated by heated repellent mosquito mats reached the peak air concentration of 36 μg/m^3^ after three hours with closed windows and 4 μg/m^3^ with open windows [20]. Considering that living indoors takes a substantial fraction of a lifetime for most people, and ingestion of foodstuffs and dermal contact with deposits of insecticide droplets or fumes on surfaces might contribute to exposure more than inhalation [21], even low concentrations of short half-life insecticides, if repeated daily and for prolonged hours, might adversely affect human health. Therefore, because of the widespread use of household insecticides, even small excess risks assume relevance from the Public Health perspective and suggest the need for further investigation on household insecticides in areas where such use is most common [16].

In the present study, we used the database of a population-based case-control study conducted in Sardinia, Italy, to investigate whether the household use of insecticides might play a role in the aetiology of lymphoma subtypes. Answering this question conveys important Public Health decisions because indoor use of insecticides not only protects against vector-borne diseases but also prevents sleep loss due to mosquito bites, which is itself a cause of adverse health effects.

## 2. Materials and Methods

### 2.1. Study Design and Participants

In 1998–2004, we conducted a population-based case-control study on the aetiology of lymphoma in Sardinia, Italy, as part of the European multicenter study EPILYMPH. Study details can be found elsewhere [22]. Briefly, in 1998–2004, 451 incident cases with a first diagnosis of lymphoma, including all B-cell and T-cell subtypes and Hodgkin lymphoma, aged 25–74 years, were identified in two participating hospitals, the A. Businco Oncology Hospital in Cagliari and the S. Francesco Hospital in Nuoro. These hospitals are referral centres for haematological malignancies over the whole region. We excluded 64 deceased patients, five who were too sick to participate, 23 we could not track down after hospital discharge, and 34 who refused participation. Therefore, the study population comprised 325 lymphoma cases (all subtypes combined). Based on the 2008 WHO classification of lymphoma [23], these included 254 B-cell lymphoma cases. Table 1 shows the frequency distribution of individual subtypes in our case series.

Eligible controls were 832 subjects randomly selected within the same time frame of case recruitment among the resident population of the study area frequency matched to cases by gender, 5-year age groups, and residence (local health unit). After excluding 17 deceased individuals, eight who were too sick to participate, 43 who we could not trace at the available address, 299 who refused participation (299/764, 39.1%), and two subjects whose residential history was not available, 465 subjects (55.6% of the eligible contacts) remained for study. The Ethical Committee of the University Hospital of Cagliari approved the study protocol (N. 148/04/C.E. of 19.11.2004). All study participants signed an informed consent form according to the Helsinki Declaration.

### 2.2. Questionnaire and Household Insecticides in Residential History

Trained interviewers administered in person to all the cases and controls a modified version of the EpiLymph questionnaire, gathering information on socio-demographic factors, lifestyle habits, such as alcohol drinking and tobacco consumption, and health, occupational, and residential history. For the six longest-held residences, the local version of the EpiLymph questionnaire also enquired about the use of household insecticides, and the frequency (days/week or month) and seasonality of use. A final question requested a free text report on the specific insecticide/s most frequently used.

### 2.3. Cumulative Exposure Assessment

Based on the average frequency and total duration of use, we calculated the lifetime days of use as a surrogate of cumulative exposure to household insecticides:

C = Σ (days/year)*_i_* × d*_i_*

where

C = cumulative exposure score in total number of days of use

*i* = *i*th residence

d = duration of use (in years).

We then categorized the lifetime days of use into quartiles of their distribution among the exposed cases and controls combined. The cumulative number of days of use calculation was repeated within each one of three time windows: before 1979, between 1979 and 2001, and from 2002 to 2004, the end of the recruitment for participation in the study. We set the cut-offs between time windows based on the prevalent type of insecticide used for exterminating insects, primarily flies and mosquitoes, on the household premises. Indoor DDT spraying was widespread in Sardinia households following its success in a 1946–1950 anti-malarial campaign [24]. Several websites refer to 1978 as the year of DDT banning in Italy. As we were unable to track the origin of these statements, we considered the year following the issue of the European Council Directive 79/117/EEC [25] as the year of discontinuing DDT use and the cut-off between the first and the second time window. In the following years, propoxur took the lead as the main ingredient of various Baygon© preparations, including the mosquito repellent mats that became popular for their easy use with electric hotplates. In 2003, SC Johnson acquired the marketing rights for Baygon© and other products, although Bayer kept manufacturing the active ingredients [26]. Coincidentally, in 2001, a paper was published raising the hypothesis of a link between household use of propoxur and the risk of childhood leukaemia [13], and the Baygon composition gradually changed to include several pyrethroids and chlorpyrifos, up to replacement [27]. Following the 8 July 2015 Federal Register Notice of Receipt of Requests to voluntarily cancel the propoxur registration, on 22 September 2015, the United States Environmental Protection Agency issued an order for propoxur cancellation [28]. At that time, the manufacturers had already eliminated propoxur in their Baygon home spray pesticides and replaced it with transfluthrin and other pyrethroids [29]. As we found notes that, in some countries, such replacements had already been taking place in 2002, we set 2002 as the cut-off between the second and the third time window.

### 2.4. Statistical Methods

The Odds Ratio (OR) and its 95% confidence interval (CI) of lymphoma (all subtypes combined), B-cell lymphoma (BCL), and its most prevalent subtypes, namely DLBCL, CLL/SLL, FL, MM, and Hodgkin’s Lymphoma (HL), associated with the use of household insecticides, were calculated using unconditional logistic regression models. All models included age, sex, education, and occupational exposure to any pesticide as the adjusting covariates. Subjects who never used household insecticides were the unexposed reference. Subjects who used household pesticides in more than one time window counted as exposed in each. Subjects who were unexposed in a time window but used household insecticides in the previous or subsequent time window were first included among the unexposed, then excluded from the dataset in a sensitivity analysis.

Also, in case of a positive association, we calculated the test for linear trend across quartiles of the total number of days of use with the Wald statistics after continuous transformation of the covariates in the logistic model. We set the two-tailed α error threshold to reject the null hypothesis at *p* < 5%. All the analyses were conducted with SPSS^®^ version 20.0.

## 3. Results

Table 2 shows the distribution of cases and controls by the demographic and exposure variables. A slightly higher proportion of refusals among the younger age classes and males resulted in minimal differences in the mean age (cases: mean age 56.0, standard deviation [*sd*] 13.9; controls: mean age 57.3, *sd* 12.9; *p* = 0.177) and gender (*p* = 0.764) by case-control status, indicating a satisfactory though partial control through the matching procedure. Education level was also comparable by case-control status (*p* = 0.997). Over the whole study population, there was no difference in the proportion of the exposed to household insecticides by case-control status (*p* = 0.086). As expected from previous reports, occupational exposure to pesticides was slightly prevalent among the cases.

Table 3 shows the risk of lymphoma, BCL and the most represented subtypes associated with the use of household insecticides over the lifetime of study participants and within the three time windows we inquired into. An increase in the risk estimates for lymphoma (all subtypes), FL, and HL associated with ever use of household insecticides in the most recent time window was generated by a small number of study subjects. After excluding from the unexposed those who had been exposed in the first and/or the second time window, the numbers became even smaller, and the risk estimates showed extreme ups and downs, which prevented any inference. There was no excess risk of lymphoma (all subtypes), BCL, and major subtypes over the lifetime, and in the other two time windows we explored. MM risk was the only subtype showing a moderate association with the use of household insecticides in the first (OR = 1.5, 95% CI 0.49–4.34) and the second (OR = 1.6, 95% CI 0.53–4.72) time windows. However, we did not observe an increasing trend with cumulative days of use in either period (*p* for trend = 0.627 and 0.203, respectively) (Table 4). Results were consistent by sex (not shown in the Tables).

In a sensitivity analysis for exposure in the first time window, we excluded from the unexposed subjects those who had been exposed in the second or third time window. In this analysis, the OR was 1.4 (95% CI 0.45–4.06) for MM with no elevation in risk for the other subtypes, lymphomas (all subtypes), or BCL combined. Only one MM case and eight controls started using household insecticides in the second time window. Therefore, we included exposure in either time windows as covariates in the regression model to adjust the respective effects. Exposure in the second time window was associated with a moderate increase in risk of BCL (OR = 1.4, 95% CI 0.43–4.46), DLBCL (OR = 1.8, 95% CI 0.42–7.77), and HL (OR = 1.5, 95% CI 0.13–16.5), while the number of MM cases who had been exposed in the second but not the first time window was too small to provide reliable results. Interestingly, among subjects who were 25 years old or less when they started using household insecticides in the second time window, the OR for MM was 0.6 (95% CI 0.10–4.05). Instead, it was 4.2 (95% CI 1.61–11.02) among those aged 26 years or older in the corresponding period.

## 4. Discussion

Our study did not find an association between exposure to household insecticides and the risk of lymphoma and its major subtypes. Dubious signals emerged of an increase in the risk of MM associated with cumulative days of use of household insecticides in the time window between 1979 and 2001, when propoxur, under the Baygon© label, was the best seller for such uses. MM risk increased significantly for users aged 26 years or older in 1979. However, the number of MM cases was too small to exclude chance as a possible determinant, and the upward trend with cumulative days of use did not reach the threshold to reject the null hypothesis. Therefore, we interpreted our findings as negative for an association while calling for further, more powerful studies with the support of more detailed exposure information.

The evidence from the existing literature is scanty and mostly related to pesticides in general or chemical families thereof and NHL. The frequency of parental use of household insecticides was associated with the risk of childhood NHL [12] and childhood leukaemia [13]. As it concerns carbamates, based on measurements from February 1999–May 2001, propoxur was among the pesticides showing the highest levels in carpet dust samples from 513 homes in four U.S. states [30]. Besides, it was one of the two most weighted contributors in a score of multiple exposures associated with an increase in NHL risk in the U.S.A. [31]. As it concerns occupational exposures in agricultural settings, carbaryl is the most frequently used carbamate insecticide. The Agricultural Health Study follow-up of cancer incidence in a large cohort of carbaryl users showed a non-significant increase in NHL risk, but did not present separate figures for MM [32]. Risk of NHL was also elevated for ever exposure to carbaryl in the pooled analysis of case-control studies conducted in Canada and the U.S.A [33]. This study also observed a tendency to an increasing risk with lifetime exposure days, although risk decreased after adjustment for concurrent exposure to other pesticides, such as Diazinon, Chlordane, Malathion, and Dieldrin [33]. The association between the agricultural use of insecticides and risk of NHL subtypes was investigates in more detail in the pooled analysis of the InterLymph case-control studies. Risk of NHL (all subtypes combined) was elevated for exposure to organophosphates, and specifically diazinon. As it concerns the carbamate insecticides, the authors could only investigates ever exposure to carbaryl: risk was elevated for FL and the T-cell lymphoma subgroup, but not MM [10]. Instead, the results of the European EpiLymph case-control study were negative for an association between carbamates and NHL risk [7]. In most pesticide studies, the analysis of MM risk was limited by the small study size or, in large studies, by the lack of occupational information. However, signals of an association with agricultural exposures were detected in the pooled analysis of InterLymph studies [34] and in the EpiLymph study [35].

In experimental studies, 2 years after the experiment started, propoxur induced severe bladder hyperplasia with neo-vascularization and carcinoma when administered at high doses in female Wistar rats but not in mice or hamsters [36]. Promoting properties, but not initiating nor complete carcinogenic properties, were observed when applied on mouse skin [37]. Still, the U.S. Environmental Protection Agency has not classified the human carcinogenicity of propoxur [38] and, so far, the International Agency for Research on Cancer has not considered it for an evaluation.

There was no excess risk of lymphoma and its most prevalent subtypes for the use of household insecticides before 1979 when DDT was most likely the only agent used for such purposes in the study area. DDT is one of the persistent organic pollutants (POP) regulated by the 2001 Stockholm Convention [39], which use the World Health Organization nonetheless permitted for indoor residual spraying against malaria [40]. With the publication of Monograph N. 113, IARC classified DDT as a probable human carcinogen (group 2A), with limited evidence of an epidemiological association with cancer of the liver, testis, and NHL and sufficient evidence for liver cancer from animal studies [5]. However, the follow-up of cancer mortality in a cohort of DDT applicators heavily exposed during a historical anti-malarial campaign that covered the whole region of Sardinia did not show an increase in mortality from cancer of the haemolymphatic tissue in the subcohort of the most severely exposed [24]. Also, a multicentre European case-control study did not find an association between the serum level of DDT residues and the risk of NHL and its subtypes DLBCL and CLL [2]. Although the exposure assessment was imprecise, our results would not support an increase in the risk of lymphoma and its most prevalent subtypes associated with the use of household insecticides in the years when DDT was in the market.

We observed an increase in risk of lymphoma (all subtypes), FL, and particularly HL for use of household insecticides from 2002 onwards, when pyrethroids became the top insecticides for household uses. The IARC Monograph Programme evaluated permethrin and deltamethrin, two major pyrethroid insecticides [41]. For both, the final evaluation was “not classifiable as for human carcinogenicity”. Also, use of permethrin did not convey an increase in mortality from neoplasms of the haemolymphatic tissue in the Agricultural Health study [42]. None of the other pyrethroids has shown an association with risk of NHL or any other cancer. On the light of the available evidence, we conclude that the increase in risk of lymphoma (all subtypes), FL, and HL we observed with the use of household insecticides from 2002 onwards was generated by chance due to the small number of the exposed study subjects.

Our study had several limitations. The first is linked to the retrospective case-control study design exposing to the possibility of recall bias. Use of household insecticides was self-reported with a binary yes/no question; a second question asked to report what product was most frequently used but most participants were unable to remember or to report other than generic definitions. Also, several products might have been available for household insecticidal treatment within each time window. We identified three time windows based on those anecdotally most frequently used in each, but we cannot ascribe with certainty the individuals’ exposure to one or another. No monitoring data were available on indoor air or biomarkers. Also, the questionnaire did not address details about the compliance with the prescription rules of indoor insecticide use, which might have been used for a score to discriminate increasing exposure levels, such as to avoid entering a room for the prescribed time after the insecticide application, to ventilate the room before entering, to avoid staying in the room during the functioning of the electric hotplate supporting the insecticide tablet, and to keep windows and doors opened while in the room after the treatment. Such information might have helped to disentangle the effect of duration from intensity of exposure.

However, self-reported frequency and duration of household exposure to pesticides was previously reported as reliable [43]. A second reason for concern is related to the multiple risk calculations we made. Besides being non-significant from the statistical perspective, the upward trend in risk of MM by cumulative days of use of household insecticides in the second time window was unexpected and likely due to chance because of the multiple comparisons we made. The small number of cases of specific BCL subtypes is the third major limitation in our study, making spurious findings more likely to occur because of the larger chance fluctuations in the risk estimates.

A substantial proportion of cases and controls did not have the information on occupational pesticide exposure. To double check whether the incomplete information on a plausible confounder affected the association between household use of insecticides and risk of lymphoma, we simulated several exposure scenarios among the cases and controls. In brief, we randomly selected among the cases and controls with missing information on occupational pesticide exposure: (a) a proportion of exposed similar to that of the cases and controls with available information; (b) a proportion of exposed 50% higher than that observed among the cases and controls with available information; (c) a proportion of exposed 50% lower than that observed among the cases and controls with available information; (d) a proportion of exposed among the cases 50% higher than that observed among the cases with available information and a proportion of exposed among the controls similar to that of the controls with available information; (e) a proportion of exposed among the controls 50% higher than that observed among the controls with available information and a proportion of exposed among the cases similar to that of the cases with available information; (f) a proportion of exposed among the cases 50% lower than that observed among the cases with available information and a proportion of exposed among the controls similar to that of the controls with available information; (g) a proportion of exposed among the controls 50% lower than that observed among the controls with available information and a proportion of exposed among the cases similar to that of the cases with available information.“ While the higher number of study subjects included in the analysis resulted in risk estimates closer to unity, in no instances the changes in the proportion of exposed among the study subjects with missing information on a plausible confounder, such as occupational exposure to pesticides, affected the risk estimate associated with use of household insecticides (not shown in the Tables). We observed a substantial decrease in the excess risk of lymphoma (all subtypes) associated with the use of household insecticides in the most recent time window. In our view, this confirmed that the excess was due to a random fluctuation of the risk estimate generated by the small number of the exposed. By analogy, the risk estimates for the lymphoma subtypes, also based on very few exposed, might have also been affected.

## 5. Conclusions

While our results are not supportive of an association between use of household insecticide and risk of lymphoma overall and BCL in particular, further investigations with a larger sample size and a detailed exposure assessment are warranted to confirm or discard the hypothesis that a mosquito-free good night sleep balances the risk of lymphoma associated to the use of household insecticides.

## Figures and Tables

**Table 1:** Frequency of the most frequently represented subtypes among the study participants.

Diagnosis	N	%
All Lymphomas	325	100
B cell lymphomas	254	78
Diffuse large B cell lymphoma	92	28
Follicular lymphoma	35	11
Chronic Lymphocytic Leukaemia/Small Lymphocytic Lymphoma	67	21
Multiple Myeloma	23	7
Other B-cell lymphoma subtypes	37	11
T cell Lymphoma	15	5
Unspecified non Hodgkin Lymphoma	24	7
Hodgkin Lymphoma	32	10

**Table 2:** Distribution of the study population by sex, age, education, and frequency of use of household insecticides.

	Cases		Controls		*p*-Value *
	N	(%)	N	(%)	
Total	325	100.0	465	100.0	
Male	188	57.8	264	56.8	0.764
Female	137	42.2	201	43.2	
Age (years)					
≤29	16	4.9	12	2.6	0.949
30–39	37	11.4	45	9.7	
40–49	43	13.2	66	14.2	
50–59	78	24.0	114	24.5	
60–69	94	28.9	139	29.9	
≥70	57	17.5	89	19.1	
Education (years)					
≤8	158	48.6	230	49.5	0.997
9–13	119	36.6	164	35.3	
≥13	48	14.8	71	15.2	
Occupational exposure to pesticides					
Yes	34	10.5	29	6.2	0.086
No	228	70.1	307	66.0	
Missing	63	19.4	129	27.8	
Lifetime days of use of household insecticides					
Never used	148	44.3	212	44.3	
Ever used	177	55.7	253	55.7	0.988
- up to 1978	166	51.1	245	52.7	0.839
- in 1979-2001	144	44.3	203	43.7	0.917
- in 2002-2004	15	4.6	23	4.9	0.845

Note: * *χ*^2^ test.

**Table 3:** Risk of lymphoma and major B-cell lymphoma subtypes associated with prolonged use of household insecticides.

Time Window for Exposure	Lymphoma (All Types) Ca/co OR (95% CI)	B-Cell Lymphoma Ca/co OR (95% CI)	DLBCL Ca/co OR (95% CI)	FL Ca/co OR (95% CI)	CLL/SLL Ca/co OR (95% CI)	MM Ca/co OR (95% CI)	HL Ca/co OR (95% CI)
Ever	177/253 0.8 (0.54–1.05)	143/253 0.9 (0.65–1.31)	52/253 1.0 (0.58–1.59)	16/253 0.6 (0.27–1.24)	36/253 1.0 (0.58–1.84)	17/253 1.3 (0.45–4.01)	15/253 0.3 (0.13–0.81)
Before 1979	166/245 0.7 (0.51–1.00)	136/245 0.9 (0.63–1.27)	49/245 0.9 (0.55–1.49)	15/245 0.6 (0.26–1.19)	35/245 1.0 (0.59–1.87)	16/245 1.5 (0.49–4.34)	13/245 0.3 (0.11–0.74)
1979–2001	144/201 0.8 (0.56–1.11)	116/201 0.9 (0.65–1.33)	42/201 0.9 (0.57–1.57)	14/201 0.6 (0.30–1.40)	27/201 0.9 (0.52–1.70)	15/201 1.6 (0.53–4.72)	15/201 0.4 (0.17–1.04)
2002–2004	15/23 1.8 (0.49–6.39)	9/23 1.1 (0.25–5.25)	1/23 -	2/23 2.6 (0.28–24.6)	2/23 1.5 (0.16–13.9)	1/23 -	4/23 11.6 (1.55–86.6)

Notes: Logistic regression model including age (continuous), sex, education, and occupational exposure to any pesticide. Abbreviations: Ca/co = cases/controls; 95% CI = 95% confidence interval; CLL/SLL = chronic lymphocytic leukemia/small lymphocytic lymphoma; DLBCL = diffuse large B-cell lymphoma; FL = follicular lymphoma; HL = Hodgkin’s lymphoma; MM = multiple myeloma; OR = Odds Ratio.

**Table 4:** Risk of lymphoma (all subtypes), B-cell lymphoma, and major B-cell lymphoma subtypes by quartiles of total days of household insecticide use in 1979–2001.

Days of Use in 1979–2001	Lymphoma (All Types) Ca/co OR (95% CI)	B-Cell Lymphoma Ca/co OR (95% CI)	DLBCL Ca/co OR (95% CI)	FL Ca/co OR (95% CI)	CLL Ca/co OR (95% CI)	MM * Ca/co OR (95% CI)	HL Ca/co OR (95% CI)
Ever used	144/201 0.8 (0.56–1.11)	116/201 0.9 (0.65–1.33)	42/201 0.9 (0.57–1.57)	14/201 0.6 (0.30–1.40)	27/201 0.9 (0.52–1.70)	15/201 1.6 (0.53–4.72)	15/201 0.4 (0.17–1.04)
≤156 days	33/56 0.6 (0.35–1.08)	29/56 0.8 (0.46–1.46)	15/56 1.2 (0.56–2.39)	3/56 0.6 (0.16–2.07)	3/56 0.3 (0.06–1.28)	3/56 1.5 (0.27–8.38)	1/56 0.1 (0.01–0.89)
157–312 days	47/41 1.3 (0.77- 2.34)	42/41 1.5 (0.89–2.66)	14/41 1.8 (0.84–3.71)	5/41 1.1 (0.33–3.39)	11/41 1.3 (0.53–3.04)	4/41 2.0 (0.37–10.7)	3/41 0.8 (0.15–4.03)
313–2002 days	32/55 0.5 (0.31–0.94)	26/55 0.7 (0.37–1.19)	10/55 0.7 (0.29–1.54)	3/55 0.3 (0.07–1.40)	4/55 0.6 (0.20–1.93)	5/55 2.6 (0.56–12.5)	4/55 0.3 (0.06–1.06)
≥2003 days	34/54 0.8 (0.45–1.40)	21/54 0.8 (0.43–1.45)	4/54 0.3 (0.10–1.19)	3/54 0.7 (0.19–2.52)	9/54 1.4 (0.62–3.28)	3/54 2.1 (0.39–11.2)	7/54 1.1 (0.30–4.17)

Note: * test for trend: *p* = 0.203. Logistic regression model including age (continuous), sex, study center, education, and occupational exposure to any pesticide. Abbreviations: Ca/co = cases/controls; 95% CI = 95% confidence interval; CLL/SLL = chronic lymphocytic leukemia/small lymphocytic lymphoma; DLBCL = diffuse large B-cell lymphoma; FL = follicular lymphoma; HL = Hodgkin’s lymphoma; MM = multiple myeloma; OR = Odds Ratio.

## Data Availability

The data presented in this study are available on request from the corresponding author. The data are not publicly available due to restrictions posed by the Ethical Committee.
